# Recent Trends in Bacteriology of Adult Patients with Chronic Rhinosinusitis

**DOI:** 10.3390/jcm8111889

**Published:** 2019-11-06

**Authors:** Doyeon Kim, Abdullah M. Assiri, Ji Heui Kim

**Affiliations:** 1 Department of Otorhinolaryngology–Head and Neck Surgery, Asan Medical Center, University of Ulsan College of Medicine, Seoul 05505, Korea; 2 Department of Otolaryngology–Head and Neck Surgery, College of Medicine, Najran University, King Abdulaziz Road, Najran 1988, Saudi Arabia

**Keywords:** microbiology, chronic rhinosinusitis, bacteria, swab, culture, endoscopic sinus surgery

## Abstract

This study aimed to identify trends in bacteria isolated from Korean adults with chronic rhinosinusitis (CRS). Enrolled were CRS patients who underwent sinus bacterial culture during endoscopic sinus surgery between 2007–2008, 2011–2012, and 2017–2018 (*n* = 510). Patients’ clinical characteristics, bacterial culture results, and antibiotic resistance were reviewed. The bacteria isolation rate was 76.3% (73.9% for CRS with nasal polyps and 82.8% for CRS without nasal polyps; *p* = 0.038). In total, 650 strains were isolated, the most common was Coagulase Negative *Staphylococci* (CNS) (28.0%), followed by *Streptococcus* species (12.2%), *Propionibacterium* species (8.0%), *Corynebacterium* species (7.5%), *Staphylococcus aureus* (6.2%), *Haemophilus* species (5.7%), *Klebsiella* species (5.1%), and *Pseudomonas aeruginosa* (4.2%). Furthermore, an analysis of the bacterial trends in the three groups showed significant increases over time for the isolation of CNS (*p* = 0.006), *Klebsiella* (*p* = 0.002), and *P. aeruginosa* (*p* = 0.007) and extended-spectrum beta-lactamase (ESBL) producing *Klebsiella* (*p* < 0.001) and *Enterobacter* (*p* = 0.007) species in terms of antibiotics resistance. This study demonstrates that the frequency of CNS, *Klebsiella*, and *P. aeruginosa* in CRS patients and the ESBL-producing *Klebsiella* and *Enterobacter* species has significantly increased in CRS patients over the last decade.

## 1. Introduction

Chronic rhinosinusitis (CRS) is an inflammatory mucosal disease of the nasal cavity and paranasal sinuses that lasts more than 12 weeks. CRS is phenotypically classified as either CRS with nasal polyps (CRSwNP) or CRS without nasal polyps (CRSsNP). The current hypothesis of CRS pathogenesis involves dysfunctional host-environmental interactions involving various exogenous pathogens and changes in the sinonasal mucosa. Although the role of bacteria as exogenous pathogens in the initial establishment of CRS remains unclear, an impaired local immune system in the sinonasal mucosa does allow colonization and overgrowth of bacteria that subsequently induce an inflammatory and immune response. Moreover, bacteria can trigger an acute exacerbation of CRS, contributing to ongoing, recalcitrant CRS [1,2].

CRS affects the quality of life. Purulent nasal discharge (anterior or posterior or both) is not only a diagnostic criterion of CRS but also one of the most common symptoms that make CRS patients uncomfortable. Although microbiome studies have reported various organisms and their important roles in the pathophysiology of CRS [3], the selection of antibiotics for infectious exacerbations in CRS patients is recommended to follow the results of bacterial culture, ideally endoscopically guided culture [1,4]. Antibiotics for CRS in South Korea have been abused (e.g., by being prescribed in the absence of culture data), fueling concerns about the emergence of atypical bacterial strains and antibiotic-resistant strains. Such problems can contribute to an increased incidence of refractory CRS after endoscopic sinus surgery (ESS). Therefore, sinus bacterial cultures during ESS are necessary to guide targeted antibiotic treatment after ESS [2,5]. Furthermore, a study of the recent bacteriological trends in CRS patients may contribute to the management of bacteriology after ESS.

We have routinely performed endoscopically guided bacterial culture from the sinus during ESS. Endoscopically guided culture is an acceptable method for obtaining bacteria cultures from patients with CRS [2,5,6,7]. The aim of this study was to investigate the bacterial strains that are frequently isolated from patients who underwent endoscopically guided bacterial culture from sinus during ESS performed within two-year periods spaced at five-year intervals over the last ten years (2007–2008, 2012–2013, and 2017–2018), changes in trends of bacteria, and their antibiotic resistance in Korean adult patients with CRS.

## 2. Materials and Methods

### 2.1. Study Population

A total of 510 adult patients (age >18 y) with CRS who underwent endoscopically guided bacterial culture from the sinus during ESS at the Asan Medical Center between January 2007 and December 2008 (*n* = 185), between January 2011 and December 2012 (*n* = 131), and between January 2017 and December 2018 (*n* = 194) were retrospectively enrolled in this study. All patients were diagnosed with CRS, according to the EPOS 2012 diagnostic criteria [1]. Each patient underwent an endoscopic exam and a computed tomography scan before ESS and was not treated with antibiotics or systemic or intranasal corticosteroids for at least four weeks before surgery. Demographic data (age, sex, current smoker), atopy status (positive result for specific IgE or skin prick test), and history of asthma were collected. According to EPOS criteria, 376 patients were diagnosed with CRSwNP, and 134 patients were diagnosed with CRSsNP. Patients who did not undergo bacterial culture during ESS or were diagnosed as fungal ball sinusitis, allergic fungal sinusitis, odontogenic sinusitis, immotile-cilia syndrome, or cystic fibrosis were excluded from this study. This study was approved by the Institutional Review Board of the Asan Medical Center, which exempted the study from requiring individual patient consent.

### 2.2. Sample Collection

Specimens were collected during ESS. The whole face, including the external nose and vestibule, was sterilized with chlorhexidine before ESS. ESS was performed by one of two experts (B.-J.L. or co-author J.H.K.). Purulent discharge in the maxillary and ethmoid sinuses was aseptically obtained using Blakesley forceps. If purulence was not observed on endoscopy, bacterial cultures were not performed during ESS. If purulence was observed on the side where surgery was performed first, bacterial culture was performed on that side. Collected specimens were placed into Thansystem (Copan Italia Spa, Brescia, Italy) collection units containing 5 mL of agar gel medium for aerobes and anaerobes and transferred to a microbiological laboratory for aerobic and anaerobic culture immediately following the procedure. Gram staining and quantitative cultures were performed. Each sample was inoculated onto plates of blood agar, MacConkey agar, and chocolate agar and then incubated at 37 °C in a 5% CO_2_ incubator for aerobes. Samples inoculated onto Brucella agar were incubated in an anaerobic atmosphere using an anaerobic chamber. Plates were examined every day. All cultured bacteria were identified based on standard microbiologic techniques [8]. Both cultured bacteria and antibiotic resistance were recorded.

### 2.3. Statistical Analyses

All analyses were performed using a statistical software package (IBM SPSS Statistics for Windows, Version 22.0. Armonk, NY: IBM Corp). The chi-squared test statistic and student’s *t*-test were used for group comparisons. A linear-by-linear association test was performed to identify increasing or decreasing trends of specific bacteria. Statistical significance was accepted for *p* < 0.05.

## 3. Results

Bacteria were isolated from 389 of 510 patients (76.3%). There were no differences between age or sex and positive culture (*p* = 0.889 and *p* = 0.835, respectively) (Table 1). CRSsNP patients showed a significantly higher isolation rate than CRSwNP patients (82.8% vs. 73.9%, *p* = 0.038). However, there were no differences in isolation rates relative to atopy, asthma, or current smoker status (*p* > 0.05 for each) (Table 2).

A total of 650 strains were isolated from the 389 patients with positive cultures, and 193 patients (49.6%) had a single isolated organism, while 196 patients (50.4%) had multiple organisms. The most frequently isolated bacteria was coagulase-negative *Staphylococcus* (CNS) (28.0%), followed by *Streptococcus* species (12.2%), *Propionibacterium* species (8.0%), *Corynebacterium* species (7.5%), *Staphylococcus aureus* (6.2%), *Haemophilus* species (5.7%), *Klebsiella* species (5.1%), *Enterobacter* species (4.3%), and *Pseudomonas aeruginosa* (4.2%) (Table 3). *Streptococci*, the second most common bacteria, were identified to the species level such as *S. pneumoniae* (4.6%), *S. constellatus* (3.4%), *S. viridans* (1.8%), *S. anginosus* (0.9%), *S. intermedius* (0.8%), and others (0.8%).

The isolation rates of specific bacteria between CRSsNP and CRSwNP patients were slightly different. *S. pneumoniae*, *S. aureus*, *Corynebacterium*, *Peptostreptococcus* species, *Haemophilus* species, *Klebsiella* species, and *Citrobacter* species were more frequent in CRSwNP patients, while *S. constellatus*, *Parvimonas micra*, and *P. aeruginosa* were more frequent in CRSsNP patients (Table 3).

In order to identify the isolated bacteria differences between eosinophilic and non-eosinophilic CRSwNP, 58 patients with CRSwNP (with positive cultures between October 2017 and December 2018) were divided into non-eosinophilic CRSwNP and eosinophilic CRSwNP as defined by eosinophilic NPs when the proportion of eosinophils exceeded 10% of total inflammatory cells [9,10]. Forty-eight strains in 36 patients with eosinophilic CRSwNP and 38 strains in 22 patients with non-eosinophilic CRSwNP were isolated. *S. epidermidis*, *Corynebacterium*, and *Enterobacter* species were more frequent in eosinophilic CRSwNP patients (each *p* < 0.05), and *Haemophilus* species, *Klebsiella* species, and *P. aeruginosa* were significantly more common in non-eosinophilic CRSwNP patients (each *p* < 0.05) (Table 4).

Next, trends of the above-listed bacteria between January 2007 and December 2018 were analyzed. First, the ten years were divided into three sections, 2007–2008, 2012–2013, and 2017–2018. Regarding the number of bacteria isolated in each period, 223 strains were identified between January 2007 and December 2008, 211 strains between January 2011 and December 2012, and 216 strains between January 2017 and December 2018. According to linear-by-linear association tests, CNS, *Klebsiella* species, and *P. aeruginosa* showed significant increasing trends (*p* = 0.006, *p* = 0.002, and *p* = 0.007, respectively), while *Propionibacterium* and *Corynebacterium* species showed significant decreasing trends (*p* < 0.001 and *p* = 0.003, respectively). However, no significant changes were found in *Streptococcus*, *S. aureus*, *Haemophilus* species, and *Enterobacter* species (*p* > 0.05 for each) (Table 5).

When trends of antibiotic resistance among frequently cultured bacteria were analyzed with linear-by-linear association tests, extended-spectrum beta-lactamase (ESBL)-producing *Klebsiella* and *Enterobacter* species were significantly increased, most notably in the most recent 2017–2018 (*p* < 0.001 and *p* = 0.007, respectively) (Table 6). The same results were obtained when analyzing the ratio of resistant bacteria per each bacterium in each period (data not shown, *p* = 0.004 and *p* < 0.001, respectively). However, methicillin-resistant *S. aureus* (MRSA) and ciprofloxacin-resistant *p. aeruginosa* were not increased.

## 4. Discussion

The overuse or misuse of broad-spectrum antibiotics may cause alterations in organisms that lead to the persistence or recurrence of sinusitis [11]. Therefore, elucidating recent trends in bacterial cultures from CRS patients is important for infection management and prevention of antibiotic resistance.

In this study on the bacteriology of Korean adult CRS patients over the past 10 years, we found that gram-positive aerobic and facultative anaerobic bacteria were the major isolates, primarily CNS (28.0%) and *Streptococcus* species (12.2%). Furthermore, gram-negative bacteria represented 29.1% of the bacterial species isolated. Among gram-negative species, the most common were *H. influenzae* (5.7%), *Klebsiella* species (5.1%)*, Enterobacter* (4.3%), and *P. aeruginosa* (4.2%). Obligate anaerobes represented 12.9%, which was primarily comprised of *Propionibacterium* (8.0%) and *Prevotella* (3.2%). These results are similar but different from previous studies. A systematic review of endoscopically derived bacterial cultures in CRS published between 1975 and 2010 showed that the composition of species was predominantly CNS (24.8%) and *S. aureus* (18.9%), followed by *H. influenzae* (9.6%), *P. aeruginosa* (7.8%), *S. pneumonia* (7.0%), *Peptostreptococcus* species (6.1%), and *Bacteroides* (6.0%) [2]. A study that enrolled 32 CRS patients in the United States between 1987 and 2004 reported that the most common aerobic and facultative bacteria were α-hemolytic *streptococci* (21.9%), Enterobacteriaceae (*Escherichia coli*, *Proteus mirabilis*, *K. pneumoniae*) (21.9%), and *S aureus* (15.6%), and the predominant anaerobic bacteria were *Peptostreptococcus* species (50.0%), *Prevotella* species (43.8%), and *Fusobacterium* species (25.0%) [12]. Bacterial culture results of biopsy specimens from anterior ethmoidal mucosa of 43 CRS patients in Germany showed that CNS (81.4%), *Corynebacterium* species (41.9%), α-hemolytic *streptococci* (20.9%), and *S. aureus* (18.6%) were the major aerobic organisms, and *Propionibacterium* (76.7%) and *Peptostreptococcus* species (25.6%) were the most common anaerobes [13].

In our study, the isolation rate of *S. aureus* (6.2%) in Korean adult CRS patients was low compared to previous studies. However, the systematic review included various compounding factors, such as the location of the specimen collection (outpatient clinic and operating room), site of culture (middle meatus and sinus), and technique (swab, aspirate, and mucosal biopsy) [2]. Another recent study involving 202 Chinese CRS patients between 2014 and 2016 reported that the most common bacteria isolated from middle meatus swab samples were CNS, *Corynebacterium*, and *S. aureus* [6]. They also collected specimens from the middle meatus before or during surgery. On the other hand, we collected specimens from the sinus in the operating room. Another reason for the discrepancy may be due to different environmental and medical factors. Other previous studies collected endoscopically guided samples from non-Asian CRS patients [12,13]. In South Korea, the isolation rate of *S. aureus* in the sinus has been reported to be less than 10%. A study in 81 CRSwNP patients who underwent ESS between 2002 and 2004 in South Korea reported that the isolation rate of *S. aureus* in the maxillary sinus of 61 adult patients was 7.8% (5 of 64 isolates), which was different from the 19.1% (13 of 68 isolates) isolation rate for middle meatus specimens [7]. Our center previously reported that the isolation rate of *S. aureus* was also 5.5% in bacterial cultures obtained from a maxillary sinus during ESS for 71 patients with CRSwNP that was diagnosed between 2007 and 2012 [8].

Other than these studies, reports of bacteriology of CRS in large patient populations are lacking in South Korea. Our study involved CRSsNP as well as CRSwNP patients and included recent data. The isolation rates for bacteria were significantly higher in CRSsNP patients than in CRSwNP patients. This result is slightly different from the results of previous studies, which reported no significant differences in isolation rates between CRSsNP and CRSwNP patients [6,13,14]. Nevertheless, the bacterial isolation rate of CRSwNP (73.9%) was notably high, as in previous studies. *S. pneumoniae*, *S. aureus*, *Corynebacterium*, *Peptostreptococcus* species, *Haemophilus* species, *Klebsiella* species, and *Citrobacter* species appear to be more common in CRSwNP patients compared to CRSsNP patients like in earlier Chinese studies [6,14]. Especially, *S. epidermidis*, *Corynebacterium* species, and *Enterobacter* species were significantly associated with eosinophilic CRSwNP and *Haemophilus* species, *Klebsiella* species, and *P. aeruginosa* with non-eosinophilic CRSwNP. Non-eosinophilic CRSwNP has been expected to be more affected by a bacterial infection and different microbiology compared to eosinophilic CRSwNP since non-eosinophilic CRSwNP is associated with Th1 and Th17 immune response but eosinophilic CRSwNP with Th2 response [15,16]. However, our results suggest that eosinophilic CRSwNP could be affected by bacteria as well as non-eosinophilic CRSwNP. Although slightly different from our results, a study in Japan, including 51 isolates of 29 patients, also demonstrated isolation rate of bacteria were high (90%) in eosinophilic CRSwNP patients, which were not different with neutrophilic CRSwNP (98%) and no differences in detected bacteria between two groups [16]. Two other previous studies showed that *S. aureus* were increased in CRSwNP with blood eosinophilia, [6] and significantly less gram-negative aerobic and facultative anaerobic bacteria were isolated from the CRS patients with blood eosinophilia [14]. Therefore, bacteria may play a role in the pathophysiology of CRSwNP and CRSsNP, regardless of eosinophilic inflammation.

We found a high prevalence of CNS in both CRSsNP and CRSwNP patients with *S. epidermidis* presenting the majority of isolated CNS. The role of CNS in the pathogenesis of CRS remains controversial as it can be frequently found in the middle meatus of healthy individuals as well as CRS patients [6,14,17]. Scant or light growth likely represents contamination, whereas moderate to heavy growth, with many WBCs on a gram stain, may be an actual infection [4]. It has been proven (using in vitro biofilm assay and a rat central venous catheter infection model) that certain strains of CNS can form biofilm [18]. Zhang et al. found that 28% of CRS patients had CNS as their only isolate, and were significantly more likely to have preoperative antibiotics but less likely to have preoperative systemic steroids and a prior ESS compared to patients with all other positive culture results. However, the Lund-Mackay CT and symptoms scores were not associated with the single result of CNS in a multiple logistic regression model [19].

A concern about increases in atypical bacteria and antibiotic-resistant organisms motivated us to investigate trends in the species composition of isolated bacteria and antibiotic resistance. When these trends over the last ten years were analyzed, CNS, *Klebsiella* species, and *P. aeruginosa* showed increasing trends that were significant. Since *Klebsiella* species and *P. aeruginosa* were not prevalent in past studies, their increase may raise awareness of the increase in gram-negative bacteria and their role in CRS; hence, antimicrobial therapy based on the culture results should be considered [20]. Moreover, we found that ESBL-producing *Klebsiella* and *Enterobacter* species have tended to increase significantly in recent years. Antibiotics have been recommended to treat these pathogens associated with acute or chronic infection [21]. The emergence of gram-negative bacteria or MRSA by empirical and repeated use of antibiotics can cause more recalcitrant CRS [22]. Acquiring an endoscopically guided culture can alleviate such problems and can lead to more successful treatments. This recommendation agrees with another study that also emphasized the importance of culture-directed antibiotic treatment to prevent antimicrobial resistance [23].

## 5. Conclusions

In bacterial cultures obtained from the sinus during ESS, even though gram-positive bacteria were the most frequently isolated strains in patients with CRS, gram-negative bacteria such as *Klebsiella* species and *P. aeruginosa* showed a significantly increasing trend. Furthermore, ESBL-producing *Klebsiella* and *Enterobacter* species appeared with an increasing trend in recent years. These findings support the current recommendation of using antibiotics targeted to positive cultures of infectious exacerbations in CRS patients, ideally with endoscopically guided cultures. We should be careful about using empirical antibiotics in patients with CRS. In addition, endoscopically guided cultures from the sinus during ESS may be helpful for the management of CRS patients by identifying pathogenic bacteria and their antimicrobial susceptibilities.

## Figures and Tables

**Table 1:** Comparison of age and sex distribution according to the culture results.

Variable	Bacterial Culture		*p*-Value
	Positive (*n* = 389)	Negative (*n* = 121)	
Age (years), mean ± S.D.	47.2 ± 17.0	46.9 ± 16.7	0.889
Male gender, number (%)	237 (60.9)	75 (62.0)	0.835

**Table 2:** Comparison of bacterial isolation rate according to the demographic data.

	Nasal Polyp		Atopy		Asthma		Current Smoker	
	Yes	No	Yes	No	Yes	No	Yes	No
No. of patients	376	134	51	459	33	477	87	423
Isolation rate, %	73.9	82.8	66.7	77.3	72.7	76.5	75.9	76.4
*p*-value	0.038		0.089		0.625		0.921	

**Table 3:** Number and percentage of bacterial isolates (*n* = 650 strains).

	Isolated Bacteria [*n* (%)]		
	Total Isolates (*n* = 650)	CRSsNP (*n* = 196)	CRSwNP (*n* = 454)
**Gram-positive aerobic and facultative anaerobic bacteria**			
Coagulase negative *Staphylococcus*	182 (28.0%)	55 (28.1%)	127 (28.0%)
*Staphylococcus epidermidis*	159 (24.5%)	45 (23.0%)	114 (25.1%)
Others	23 (3.5%)	10 (5.1%)	13 (2.9%)
*Streptococcus* species	79 (12.2%)	32 (16.3%)	47 (10.4%)
*Streptococcus pneumoniae*	30 (4.6%)	6 (3.1%)	24 (5.3%)
*Streptococcus constellatus*	22 (3.4%)	14 (7.1%)	8 (1.8%)
*Streptococcus viridans*	12 (1.8%)	5 (2.6%)	7 (1.5%)
*Streptococcus anginosus*	6 (0.9%)	3 (1.5%)	3 (0.6%)
*Streptococcus intermedius*	5 (0.8%)	4 (2.0%)	1 (0.2%)
*Streptococcus group C*	1 (0.2%)	0 (0.0%)	1 (0.2%)
*Streptococcus group F*	1 (0.2%)	0 (0.0%)	1 (0.2%)
*Streptococcus group G*	1 (0.2%)	0 (0.0%)	1 (0.2%)
*Streptococcus pseudopneumoniae*	1 (0.2%)	0 (0.0%)	1 (0.2%)
*Corynebacterium*	49 (7.5%)	6 (3.1%)	43 (9.5%)
*Staphylococcus aureus*	40 (6.2%)	10 (5.1%)	30 (6.6%)
*Parvimonas micra*	21 (3.2%)	13 (6.6%)	8 (1.8%)
*Peptostreptococcus* species	18 (2.8%)	2 (1.0%)	16 (3.5%)
Others			
*Peptoniphilus asaccharolyticus*	7 (1.1%)	2 (1.0%)	5 (1.1%)
*Enterococcus*	4 (0.6%)	0 (0.0%)	4 (0.9%)
*Bacillus*	2 (0.3%)	1 (0.5%)	1 (0.2%)
*Micrococcus* species	1 (0.2%)	0 (0.0%)	1 (0.2%)
**Gram-positive obligate anaerobic bacteria**			
*Propionibacterium* species	52 (8.0%)	10 (5.1%)	42 (9.3%)
*Clostridium* species	3 (0.5%)	1 (0.5%)	2 (0.4%)
**Gram-negative aerobic and facultative anaerobic bacteria**			
*Haemophilus* species	37 (5.7%)	6 (3.1%)	31 (6.8%)
*Haemophilus influenzae*	33 (5.1%)	6 (3.1%)	27 (5.9%)
Others	4 (0.6%)	0 (0.0%)	4 (0.9%)
*Klebsiella* species	33 (5.1%)	7 (3.6%)	26 (5.7%)
*Klebsiella aerogenes*	16 (2.5%)	3 (1.5%)	13 (2.9%)
*Klebsiella pneumoniae*	13 (2.0%)	3 (1.5%)	10 (2.2%)
*Klebsiella oxytoca*	4 (0.6%)	1 (0.5%)	3 (0.7%)
*Enterobacter* species	28 (4.3%)	9 (4.6%)	19 (4.2%)
*Citrobacter* species	6 (0.9%)	0 (0.0%)	6 (1.3%)
Others			
*Eggerthella lenta*	3 (0.5%)	3 (1.5%)	0 (0.0%)
*Escherichia coli*	3 (0.5%)	1 (0.5%)	2 (0.4%)
*Achromobacter xylosoxidans*	2 (0.3%)	1 (0.5%)	1 (0.2%)
*Serratia*	2 (0.3%)	1 (0.5%)	1 (0.2%)
*Morganella morganii*	2 (0.3%)	1 (0.5%)	1 (0.2%)
*Campylobacter rectus*	1 (0.2%)	0 (0.0%)	1 (0.2%)
*Eikenella corrodens*	1 (0.2%)	1 (0.5%)	0 (0.0%)
**Gram-negative obligate aerobic bacteria**			
*Pseudomonas aeruginosa*	27 (4.2%)	14 (7.1%)	13 (2.9%)
*Moraxella*	5 (0.8%)	2 (1.0%)	3 (0.7%)
*Acinetobacter*	1 (0.2%)	0 (0.0%)	1 (0.2%)
*Neisseria subflava*	1 (0.2%)	0 (0.0%)	1 (0.2%)
*Stenotrophomonas maltophilia*	1 (0.2%)	1 (0.5%)	0 (0.0%)
**Gram-negative obligate anaerobic bacteria**			
*Prevotella* species	17 (2.6%)	11 (5.6%)	6 (1.3%)
*Fusobacterium* species	4 (0.6%)	4 (2.0%)	0 (0.0%)
*Bacteroides*	2 (0.3%)	2 (1.0%)	0 (0.0%)
*Porphyromonas* species	1 (0.2%)	0 (0.0%)	1 (0.2%)

CRSsNP, chronic rhinosinusitis without nasal polyps; CRSwNP, chronic rhinosinusitis with nasal polyps.

**Table 4:** Comparison of the isolated bacteria in patients with non-eosinophilic and eosinophilic chronic rhinosinusitis with nasal polyps (CRSwNP) between October 2017 and December 2018.

Organisms	Eosinophilic CRSwNP (*n* = 48 Strains) (%)	Non-eosinophilic CRSwNP (*n* = 38 Strains) (%)	*p*-Value
*Staphylococcus epidermidis*	46.0	26.0	0.003
*Streptococcus* species	8.0	16.0	0.082
*Corynebacterium* species	12.9	0.0	<0.001
*Staphylococcus aureus*	8.0	5.0	0.390
*Haemophilus* species	0.0	10.9	0.001
*Klebsiella* species	21.0	37.0	0.013
*Enterobacter* species	4.0	0.0	0.043
*Pseudomonas aeruginosa*	0.0	5.0	0.024

**Table 5:** Trends of frequently cultured bacteria over time.

Organisms	2007–2008 (*n* = 223 Strains) (%)	2012–2013 (*n* = 211 Strains) (%)	2017–2018 (*n* = 216 Strains) (%)	*p*-Value
CNS	21.5	29.4	33.3	0.006
*Streptococcus* species	11.2	10.0	15.3	0.196
*Propionibacterium* species	12.1	11.8	0.0	<0.001
*Corynebacterium* species	11.2	7.6	3.7	0.003
*Staphylococcus aureus*	8.1	4.3	6.0	0.364
*Haemophilus* species	5.8	3.8	7.4	0.484
*Klebsiella* species	4.0	0.5	10.6	0.002
*Enterobacter* species	6.3	2.8	3.7	0.181
*Pseudomonas aeruginosa*	2.7	1.9	7.9	0.007

**Table 6:** Trends in the antibiotic resistance of frequently cultured bacteria over time.

Organism Antibiotic Resistance	2007–2008 (*n* = 223 Strains) *n* (%)	2012–2013 (*n* = 211 Strains) *n* (%)	2017–2018 (*n* = 216 Strains) *n* (%)	*p*-Value
*Streptococcus* species				
Amoxicillin/clavulanate	0 (0.0%)	0 (0.0%)	2 (0.9%)	0.082
Macrolide ^a^	8 (3.6%)	10 (4.7%)	13 (6.0%)	0.233
*Staphylococcus aureus*				
Methicillin	2 (0.9%)	1 (0.5%)	2 (0.9%)	0.977
*Haemophilus* species				
Ampicillin	8 (34.8%)	1 (0.5%)	10 (4.6%)	0.532
*Klebsiella* species				
ESBL	1 (0.4%)	1 (0.5%)	16 (7.4%)	<0.001
Ciprofloxacin	1 (0.4%)	0 (0.0%)	0 (0.0%)	0.229
*Enterobacter* species				
ESBL	0 (0.0%)	6 (2.8%)	8 (3.7%)	0.007
Ciprofloxacin	0 (0.0%)	0 (0.0%)	0 (0.0%)	N/A
*Pseudomonas aeruginosa*				
ESBL	0 (0.0%)	0 (0.0%)	0 (0.0%)	N/A
Ciprofloxacin	0 (0.0%)	0 (0.0%)	2 (0.9%)	0.082

N/A, not available; ESBL, extended-spectrum beta-lactamase; ^a^ Macrolide includes azithromycin, erythromycin, and clarithromycin.
